# Temporal dynamics between the whisker sensorimotor cortex and the olivocerebellar module in awake mice

**DOI:** 10.3389/fncel.2026.1781469

**Published:** 2026-09-30

**Authors:** G. Cheron, A. Caballero-Tapia, D. Ristori, J. Márquez-Ruiz, A. M. Cebolla, L. Ris

**Affiliations:** 1 Department of Neuroscience, Research Institute for Biosciences, Université de Mons, Mons, Belgium; 2 Movement Neurophysiology Lab NeuroMove, Neuroscience Institute, Universite Libre de Bruxelles, Brussels, Belgium; 3 Brain Stimulation translational Laboratory, Department of Physiology, Anatomy and Cell Biology, Pablo de Olavide University, Seville, Spain

**Keywords:** cerebellum, deep cerebellar nuclei, electrical stimulation, Golgi cells somatosensory and motor cortex, inferior olive, Purkinje cells, whisker

## Abstract

**Introduction:**

The precise timing of the long-range bidirectional connections between the cerebral cortex and the cerebellum remains a critical factor in elucidating their respective and integrated functions. The key role of the cerebellum in both motor and non-motor domains is exerted through numerous cerebro-cerebellar closed loops that sustain different forms of motor coordination, in which the response timing of the various types of cerebellar neurons may constitute a significant component of neural computation.

**Methods:**

In this study, we investigated the cerebral sensorimotor cortex and the corresponding cerebellar region (Crus I) of the murine whisker system during electrical stimulation of the whisker pad. In addition, the firing of Golgi cells, Purkinje cells (PC), deep cerebellar nuclei (DCN) neurons, and inferior olive (IO) neurons was analyzed during electrical stimulation of the whisker pad (Wh), the somatosensory (wS1) and motor (wM1) cortices.

**Results:**

We demonstrated that, although peripheral Wh stimulation evoked cortical, cerebellar and IO responses at the same early latency (~3 ms), the transmission within the olivocerebellar circuit was slower when the signal was elicited by wS1 stimulation than by peripheral input, and even slower when wM1 was stimulated.

**Discussion:**

We discussed the different possibilities regarding the transmission of the efference copy and the forward dynamic model within these circuits, and we speculated that the relative slowing of wM1 input into the olivo-cerebellar circuit reflects the increased computational demand associated with the elaboration of the final motor command.

## Highlights

- The electrical stimulation of the whisker pad induced rapid (3 ms) activation of the cerebellum (Crus I) and the sensory cortex (wS1).
- The electrical stimulation of the whisker pad recruited in a successive timing, the Golgi cells (2.9 ms), the simple spike (SS) of the PC (3.5 ms), the inferior olive (IO neurons (3.6 ms), the deep cerebellar (DCN) neurons (4.7 ms), and the CS (CS) of the PC (11.3 ms) of the Crus I-II.
- The electrical stimulation of wS1 recruited the same cerebellar neurons, but with another timing, the Golgi Cell (5.7 ms), the SS of the PC (7.4 ms), the DCN neurons (5.6 ms), and the CS of the PC (17.1 ms) of the Crus I-II and the IO neurons (6.0 ms).
- The electrical stimulation of the motor cortex (wM1) was far less efficient at recruiting, over a longer time frame, the SS of the PC (9.6 ms), the CS of the PC (23 ms), the DCN neurons (16.5 ms) and the IO (7.8 ms).
- It was suggested that the timing of the fastest transmission of the whisker peripheral input into the cerebellar circuit is temporally integrated into the slower input from wS1 and wM1, offering multiple integrated temporal dynamic frames for the expression of the forward dynamic model of the whisker movement.

## 1 Introduction

The execution of a skilled action requires precise motor planning supported by distributed neural circuits interconnected through long-range communication. Efficient sensorimotor integration depends critically on the temporal coordination of neuronal activity, particularly spike timing between interconnected regions (Buzsáki and Vöröslakos, 2023). Among these circuits, interactions between the sensorimotor cortex and the cerebellum are increasingly recognized as essential, yet the precise timing and integration of feedback and feedforward signals within the cortico-olivo-cerebellar loop remain incompletely understood (Welsh et al., 1995; Welsh, 2002; Lindeman et al., 2021; Boven and Cerminara, 2023; Boven et al., 2023).

Characterizing the function of a neuronal pathway requires accurate measurement of transmission delays across its relays since the initial sensory stimulation. Precise measurement of the different latencies in the eye-velocity traces during vestibulo-ocular learning was able to differentiate modifiable and non-modifiable pathways involved in oculomotor learning (Lisberger, 1984). In the same way, it was after having determined with great precision the relationships between the timing of the complex spike (CS) emitted by the Purkinje cells (PC) situated in the different cerebellar folia and the respective distances from the inferior olive (IO; Sugihara et al., 1993) that the discovery of isochronicity of CS conduction allowed LLinás to develop his cerebellar motor timing theory (Llinás, 2011).

Recent evidence supports the role of the cerebellum in sensorimotor coordination (Romano et al., 2022), prediction (Romano et al., 2025) and learning (Carey and Lisberger, 2002; Fernández Santoro et al., 2025), with neuronal coding relying in part on spike timing. In these complex processes, different types of neurons can transmit information based on the timing of single units representing a significant part of the neural code (Chaisanguanthum et al., 2014; Payne et al., 2019; Liu et al., 2025; Tang et al., 2022; Binda et al., 2023). Given the dense bidirectional connectivity between the cortex and the cerebellum (Ramnani, 2006; D’Angelo and Casali, 2012; Tremblay et al., 2019; Müller et al., 2023), the timing of their interactions is likely to be a key determinant of integrated function. Here, we investigated these dynamics in the murine whisker system, a well-established model of active sensing.

In rodents, the primary whisker somatosensory cortex (wS1) and motor cortex (wM1) form a part of a distributed network controlling rhythmic whisking behavior in which the brainstem occupies a hierarchical position (Moore et al., 2013; Petersen, 2019). In this context, inhibitory neurons of the vibrissa intermediate reticular nucleus (vIRtPV) in the brainstem form a network oscillator that is tightly interconnected with the motor cortex and the deep cerebellar nuclei (DCN), among others (Takatoh et al., 2022). The vIRtPV neurons fired tonically at rest and switched to a rhythmic bursting at the onset of whisking, a transition due to an inhibitory input. When the vIRtPV neurons were blocked, the rhythmic whisking was eliminated resulting in sustained vibrissae protraction (Takatoh et al., 2022). While brainstem circuits can generate whisking autonomously, cortical and cerebellar inputs contribute to modulation, learning, and predictive control. Notably, cerebellar PC activity (Chen et al., 2017; Chen et al., 2016) is temporally linked to whisker movements, and cerebellar nuclei may receive both inhibitory and excitatory inputs that shape motor output timing (Zhai et al., 2024).

Whiskers act as mobile sensors (Elbaz et al., 2023), scanning the immediate environment to provide textural and spatial information essential for nocturnal navigation and survival. PC in cerebellar Crus I fire in phase with spontaneous whisking, with simple spike (SS) activity typically preceding whisking onset by about 30–40 ms (Chen et al., 2016). The DCN recruitment latencies differ for protraction vs. retraction (~25 ms vs. ~8 ms), suggesting that DCN neurons can sometimes activate before PC SS firing, particularly for protraction (Zhai et al., 2024).

Whisking operates at frequencies up to ~10 Hz, requiring rapid processing across sensorimotor loops. In this context, Bauer et al. (2022) showed that the resonant frequency at 8 Hz corresponds to the cerebellar-whisker circuitry producing rhythmic whisking. The electrical stimulation of the whisker pad at 8 Hz was also the preferred frequency for inducing cerebellar plasticity (Márquez-Ruiz and Cheron, 2012). Interestingly, O’Connor et al. (2002) demonstrated that the cerebellar local field potentials (LFPs) were centered at ~ 8 Hz during whisking pauses. They decreased during the onset of whisking, while the wS1 LFP power increased during whisking.

In response to whisker stimulation, the development of an adequate motor response does not only depend on the connections between the wS1 and wM1 but on many other regions of the nervous system (Bosman et al., 2011), including the basal ganglia and the cerebellum, which play an important role not only in terms of executive motor control but also for adaptation and behavioral learning (Bosman et al., 2010; Lindeman et al., 2021). Elbaz et al. (2023) demonstrated that peripheral reafference was not required for trained rats to maintain a single vibrissa in position, and that the motor cortex was necessary only in the absence of peripheral reafference. This position control is consistent with the internal forward model concept (Wolpert et al., 1995, 1998) operating in both open-and closed-loop modes to maintain positional stability. Tanaka et al. (2020) reviewed neural evidence indicating that current outputs from the DCN can predict future cortical outputs transmitted via mossy fibers through the pontine nucleus to the cerebellum. This suggests that DCN neurons may be activated by peripheral sensory input prior to activation by cortical input.

The sensory input originating from the whisker pad is not only transmitted via the trigeminal nucleus to wS1 but also to the cerebellum and the inferior olive (IO; Bosman et al., 2011). In addition, in response to the same peripheral input, wS1 is also able to send a cortical signal to the olivo-cerebellar system, which, in turn, elaborates the cerebellar output signal toward the cerebral cortex via the thalamic nucleus (Proville et al., 2014). The transmission timing of the output signal originating in wS1 to the cerebellum is crucial for understanding the possible contribution of this signal to the type of motor control involved.

Proville et al. (2014) demonstrated that labeled wS1 and wM1 terminals in the pontine nucleus were spatially segregated, highlighting the importance of the uniqueness of the sensory-motor convergence occurring in the cerebellum. The same authors also reported, with tetrode recordings, the di-synaptic recruitment of Golgi cells and PCs of the Crus I. To our knowledge, two important elements remain to be explored; (1) the firing behavior of the DCN neurons and (2) the possible activation of the IO during the electrical stimulation of the whisker pad and the stimulation of wS1 and wM1.

In the present study, we used single-pulse electrical stimulation of the whisker pad, wS1, and wM1, to compare activation latencies across PCs, DCN neurons, Golgi Cells, and IO neurons. Based on forward dynamic model frameworks we hypothesized that sensory reafference is processed earlier than motor efference copy signals within cerebellar circuits.

Our results reveal that although the peripheral stimulus arrive simultaneously in the cerebral sensorimotor cortex and in the cerebellum, wS1 and wM1 stimulation evoked the successive recruitment of the olivo-cerebellar loop working at a different timing suggesting a specific functional role of this pathway in processing distinct sources of sensory and cortical information. This temporal organization supports a multi-timescale framework for cerebellar forward modeling in which the IO may generate multiple, temporally shifted error signals that compare predicted sensory consequences with incoming reafferent information at successive stages of the olivo-cerebellar loop.

## 2 Methods

### 2.1 Animal care

Experiments were performed in 20 8-month-old male mice in accordance with the European Union directive 2010/63/EU on animal experimentation and with the authorization of the Ethics Committee of the University of Mons. During the 24-h postoperative period, warmth and free access to high-energy liquids were provided. Every effort was made to minimize the number of animals and their discomfort. We used quartz–platinum/tungsten microelectrodes (1.2–3 MΩ) in a 7-channel Eckhorn microdrive (Thomas Recordings©, Giessen, Germany). All measures of impedance were made with a 1 kHz sinusoidal current and checked throughout the recording session. In the present study, the exploration was made with one or two microelectrodes separated from 250 μm (outer and shaft diameters of 80 μm and 25 μm, respectively). The microelectrode was mounted inside a stretched elastic rubber tube to enable proper positioning via DC-micromotors (resolution of 0.27 μm). To avoid unnecessary stress for the animals and movement artifacts, recording sessions were performed in a quiet room when the animals were awake and calm. The alertness level was monitored by assessing the maintenance of whisking activity during the recording session.

### 2.2 Surgical preparation

The mice were prepared for chronic recordings of LFPs and PC single-unit activity. They were anesthetized with xylido-dihydrothiazin (Rompun©, Bayer, 10 mg/kg) and ketamine (Ketalar©, Pfizer, 100 mg/kg), and an additional dose of xylido-dihydrothiazin (3 mg/kg) and ketamine (30 mg/kg) was administered when marked increases in respiration or heart rate were observed during the procedure. A local anesthetic (0.5 mL of 20 mg/mL lidocaine and adrenaline [1:80000, Xylocaine©, Astra Zeneca]) was injected subcutaneously during the soft tissue removal. During surgery, two small bolts were cemented perpendicular to the skull to immobilize the head during the recording sessions, and a silver reference electrode was placed on the surface of the parietal cortex. To allow access to the vermis and the Crus I and II areas as well as the deep nucleus in the cerebellum, an acrylic recording chamber was made around a posterior craniotomy (2 × 3 mm) and covered with a thin layer of bone wax (Ethicon©, Johnson & Johnson).

### 2.3 Single-unit recordings in alert mice

Twenty-four hours after the anesthesia, alert mice were restrained for the recording session. For some of them, recordings were performed during two consecutive days. The dura was removed over the cerebellum to expose the tissue in the recording chamber. Given the critical effect (Zhou et al., 2014) of the localization of the PC recording in zebrin + or zebrin- rostrocaudal bands, the specific recording place was kept in the same type of zebrin zone for all the recording sessions. The SS and CS recordings were thus performed in the PC layer of the Crus I-II in zebrin+, and in the DCN (interposed nucleus) following stereotaxic coordinates relative to Bregma: caudal 6.0–6.5 mm, lateral 3.0–3.4 mm) at a depth of 150–2000 μm (Brown and Raman, 2018). The inferior olive (IO) neurons were recorded at the stereotaxic coordinates relative to Bregma caudal 7.2 mm, lateral 0.25 mm left, at a depth of 4,500–5,500 μm. Electrical stimuli were monophasic constant rectangular current, 0.2 msec in duration and applied through a 0.1 Mohm bipolar tungsten electrode implanted in wS1 and wM1 at Bregma caudal 0.5 mm, lateral 3.2 mm left and rostral 1.4 mm, lateral 1.1 mm (left), respectively (Ferezou et al., 2007). The depth of the electrodes was also noted, and electrolytic lesions were made on the top of the cerebellum, 1 mm above the recording site in the IO and inside of the IO at the appropriate stereotaxic coordinates during a last track.

Whisker regions were electrically stimulated with a pair of small cutaneous needles inserted under the skin (inter-electrode distance 3–4 mm). Electrical stimulation consisted of a single square pulse, 0.2 ms in duration and 2 mA current intensity, delivered by an isolation unit (Isoflex, AMPI, Israel) connected to an analog pulse generator (Master 8, AMPI, Israel). The amplitude of the current was adjusted to avoid overt movements and animal discomfort.

### 2.4 Recording of the whisker movement

The movement of the whisker was recorded by means of a home-made system using the A3515/16 BiCMOS linear Hall-effect sensors measuring the displacement of a micro-magnet of 0.003 g (1.0 mm diameter and 0.5 mm height), glued onto one whisker on the contralateral side of the whisker stimulation. The onset latency of the movement was automatically detected by a Python script as the value exceeding the baseline mean by three standard deviations.

### 2.5 Identification of the neuronal units

For each spike type analyzed, the latency of the evoked recruitment elicited by the various electrical stimulation protocols was determined only after confirming, through the superimposition of isolated spike waveforms, that the responses were consistently time-locked with a jitter of less than 0.5 ms to the stimulus and did not correspond to spontaneous discharges occurring randomly in relation to the stimulation.

The identification of the different isolated neurons was based on the following criteria: For PCs, the criteria included a resting SS firing frequency ranging from 20 to 100 Hz with a average ISI of 12–25 ms, as well as the presence of CSs occurring at a resting frequency of 0.5–1.0 Hz and followed by a pause in SS firing (Cheron et al., 2004; Servais et al., 2004; Arancillo et al., 2015; Warnaar et al., 2015). For Golgi cells, identification was based on their location within the granular layer in close proximity to the PC layer, together with a somewhat irregular, relatively low firing frequency (<12 Hz; mean ISI of approximately 0.2 s), large-amplitude spikes with either positive–negative or negative–positive waveforms, and the absence of spontaneous burst firing at rest (Vos et al., 1999; Simpson et al., 2005; Barmack and Yakhnitsa, 2008). For DCN neurons, identification relied on stereotaxic coordinates (Paxinos and Franklin, 2001), previously validated by our group through neuronal activation of the red nucleus (Cheron et al., 2018), as well as on the di-to triphasic spike waveform and a relatively regular firing frequency around 50 Hz.

To avoid unnecessary stress for the animals and movement artifacts, recording sessions were performed in a quiet room when the animals were awake and calm. The alertness level was monitored by assessing the maintenance of whisking activity during the recording session. We used quartz–platinum/tungsten microelectrodes (1.2–3 MΩ) in a seven-channel Eckhorn microdrive (Thomas Recordings©, Giessen, Germany). All measures of impedance were made with a 1 kHz sinusoidal current and checked throughout the recording session. In the present study, the exploration was made with one or two microelectrodes separated from 250 μm (outer and shaft diameters of 80 μm and 25 μm, respectively). The microelectrode was mounted inside a stretched elastic rubber tube to enable proper positioning via DC-micromotors (resolution of 0.27 μm).

### 2.6 Histological control recording of the whisker movement

Before inserting the electrode for the induction of electrolytic lesions, the electrode tip was immersed in a dye solution containing 2.5 mg/mL DiI (Tetramethylindocarbocyanine Perchlorate; D282, Invitrogen™) dissolved in ethanol, following the Lipophilic Tracers protocol. The stable fluorescence of DiI enabled precise identification of electrode tracts with minimal lateral diffusion. This method, which requires no additional tissue processing beyond standard perfusion and sectioning procedures, helps preserve tissue integrity for subsequent analyses (DiCarlo et al., 1996). At the end of the *in vivo* experimental procedures, the animals underwent a perfusion and tissue preservation protocol. They were first anesthetized in an isoflurane chamber (3% Iso-Vet), followed by a lethal intraperitoneal injection of ketamine (200 mg/kg). Standard intracardiac perfusion was then performed using 4% paraformaldehyde (PFA). The brains were removed, post-fixed in 4% PFA for 48 h, dehydrated, and embedded in paraffin blocks. Sagittal sections (5 μm thick) were cut using a microtome and stained with Cresyl Violet. Fluorescence imaging was performed using a Nikon Ti-E inverted fluorescence microscope equipped with a Perfect Focus System (PFS) and a Lumencor illumination system (Nikon Corporation, Tokyo, Japan). DiI fluorescence was visualized using an excitation wavelength of 549 nm and an emission wavelength of 565 nm.

### 2.7 Data analysis and statistical methods

All recordings were performed with a bandwidth of 0.01 KHz to 10 KHz. Single units recordings were high-pass filtered at 100 Hz. All recordings were stored digitally on a computer after conversion with an analog-digital converter (Power 1,401, CED©, Cambridge, UK). The recorded data were digitized continuously at 20 kHz. Off-line analysis and illustrations were performed with Spike2 CED software *(CED©).*

The electrophysiological data are reported as mean ± SD and illustrated in box plots. The statistics were performed with the software R version 4.5.0. The level of significance was set at *p* < 0.05. To avoid pseudoreplication, the mouse was considered the independent biological unit in all analyses, irrespective of the recorded variable, including neuronal responses from the different cell populations, local field potentials (LFPs), and whisker-movement measurements. When several neurons, trials, or repeated measurements were obtained from the same mouse under the same experimental condition, these values were first averaged within that animal, and statistical analyses were subsequently performed on the resulting mouse-level values. Paired analyses included only animals represented in all compared conditions. The normality of paired mouse-level differences was assessed using the Shapiro–Wilk test. Two-condition comparisons were performed using two-sided paired-samples t-tests when the normality assumption was met and exact or permutation-based Wilcoxon signed-rank tests when it was not; non-parametric tests were also used as sensitivity analyses when sample sizes were small. Experiments involving three repeated conditions were analyzed using repeated-measures ANOVA, with Greenhouse–Geisser correction where appropriate, or the Friedman test for non-parametric data. Factorial designs were assessed using repeated-measures ANOVA to test the relevant main effects and interactions. Significant omnibus effects were followed by planned paired comparisons, with *p* values adjusted for multiple testing using the Holm procedure. Results are reported with two-sided *p* values, 95% confidence intervals, and appropriate effect-size measures, including paired Cohen’s (d_z), matched-pairs rank-biserial correlation, partial eta-squared ((\eta_p^2)), or Kendall’s (W), as applicable.

## 3 Results

### 3.1 wS1 and crus I LFP induced by whisker pad stimulation

To compare the activation latencies of wS1 and the cerebellar cortex regarding the same sensory information, isolated electrical stimulation of the whisker pad was performed. The inversion of the first negative LFP component of wS1 (Figure 1A) suggests that this activity corresponds to the initial activation of wS1, while the activation of the cerebellar cortex identified by the peak of the N2 component (Figure 1B). At the animal level, mean LFP latency was 3.1 ± 0.1 ms in wS1 and 2.9 ± 0.2 ms in the cerebellar cortex (Figure 1C). The mean paired difference was 0.19 ms (95% CI: −0.059 to 0.440 ms). Although wS1 latency was numerically longer in four of the five animals, the difference was not statistically significant (paired-samples t-test: t(4) = 2.12, *p* = 0.101; Cohen’s dz. = 0.95). An exact Wilcoxon signed-rank test yielded the same conclusion (W = 1, *p* = 0.125). This means that the earliest wS1 and cerebellar LFP responses both occurred within an early post-stimulus window of approximately 3 ms. No statistically significant difference was detected between their latencies. But they do not establish temporal equivalence or strictly simultaneous activation.

**Figure 1:**
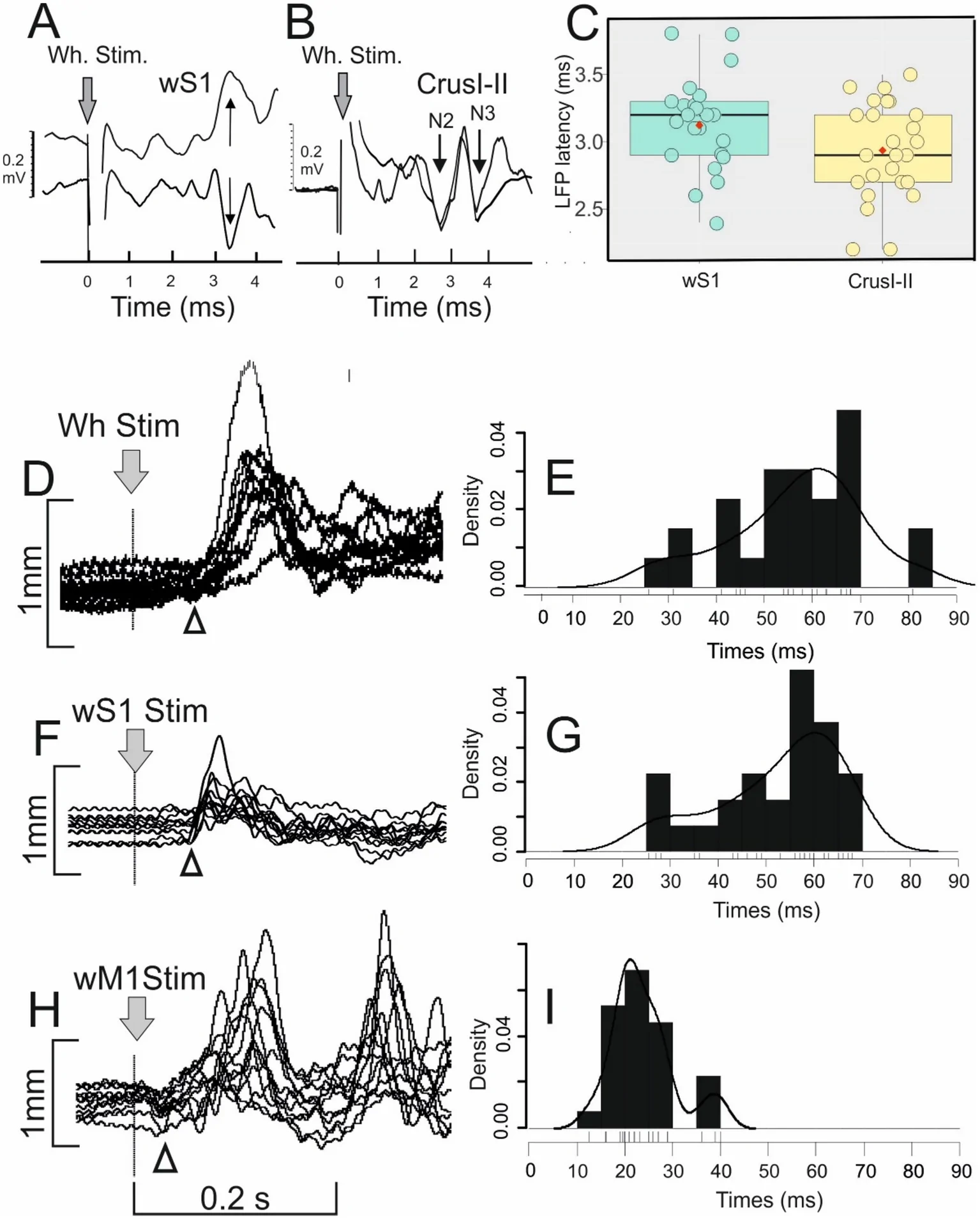
Early cortical and cerebellar responses and whisker movements evoked by electrical stimulation. **(A)** Local field potentials (LFPs) recorded along the same electrode track in the whisker primary somatosensory cortex (wS1) during single-pulse whisker pad stimulation (Wh Stim; gray arrow). The polarity inversion of the first negative component indicates that the electrode tip crossed the center of the cortical current generator and was used to estimate the earliest cortical activation. **(B)** Representative cerebellar LFP recorded in Crus I–II at the Purkinje cell (PC) layer following whisker pad stimulation. The N2 and N3 negative components are indicated by arrows. **(C)** Box plots comparing the latency of the first cortical LFP inversion in wS1 (blue) and the latency of the cerebellar N2 component recorded in Crus I–II (yellow). These LFPs correspond to the average of 10 successive stimulations performed 5 times in 5 mice and for each condition. **(D,F,H)** Superimposed whisker protraction movements evoked by electrical stimulation of the whisker pad **(D)**, wS1 **(F)**, or whisker motor cortex (wM1; **H**). Gray arrows indicate stimulus onset. Open triangles indicate movement onset. **(E,G,I)** Corresponding distributions of whisker movement onset latencies for whisker pad **(E)**, wS1 **(G)**, and wM1 **(I)** stimulation. Whisker displacement was measured with a Hall-effect sensor coupled to a miniature magnet attached to a whisker. A bin width of 5 ms was used for the histograms **(E–I)**.

### 3.2 Whisker movements induced by whisker pad, wS1 and wM1 stimulation

The electrical stimulation of the whisker (Wh) pad, wS1 and wM1 evoked small protraction movements (less than 2 mm) of the whisker (Figures 1D–I). Movement-onset latency differed significantly among stimulation conditions (Greenhouse–Geisser-corrected repeated-measures ANOVA: *F*(1.60, 6.41) = 55.10, *p* = 1.20 × 10^−4^, partial η^2^ = 0.932). Mean animal-level latencies were 56.5 ± 6.4 ms following whisker-pad stimulation, 52.3 ± 6.4 ms following wS1 stimulation, and 24.0 ± 5.1 ms following wM1 stimulation. Holm-corrected paired comparisons showed that movement latency following wM1 stimulation was significantly shorter than following whisker-pad stimulation (mean difference: 32.5 ms, 95% CI: 21.2–43.7 ms; t(4) = 8.04, adjusted *p* = 0.00260) and wS1 stimulation (mean difference: 28.3 ms, 95% CI: 21.1–35.4 ms; t(4) = 11.04, adjusted *p* = 0.00115). No significant difference was detected between whisker-pad and wS1 stimulation (t(4) = −1.26, adjusted *p* = 0.275).

### 3.3 Recruitment of olivo-cerebellar neurons by wS1 and wM1 electrical stimulation

wS1 single pulse stimulations were applied through implanted bipolar electrodes during the stable recording of 70 PCs, 71 DCN neurons, 40 Golgi cells, and 42 inferior olive (IO) neurons. Among these neurons, 29 PCs, 28 DCN neurons, 25 Golgi cells, and 38 IO neurons were responding to the single pulse stimulation of wS1. Among these neurons, the stimulation of wM1 was compared to wS1 on 12 PCs, 9 DCN and 17 IO neurons. Less than 50% of these neurons were recruited by wM1.

### 3.4 Recruitment of PC

Figure 2 illustrates the recruitment by wS1 stimulation of the SS-PC firing at the short latency of about 3.6 ms where two single SS accross four superimposed trials were evoked with a jitter smaller than 0.5 ms. This latency was the shortest latency recorded among the 29 PCs. The histogram of Figure 2D illustrates the variety of firing responses of 10 different PCs. These cells were well isolated and identified as PCs by the presence of CSs and the related pause in SS firing (as illustrated in Figure 3E). PC SS frequency increased from 100.06 ± 39.66 Hz before wS1 stimulation to 341.0 ± 151.6 Hz after stimulation. Frequency increased in each of the 12 mice. The mean paired increase was 240.9 Hz (95% CI: 157.03–324.86 Hz) and was statistically significant (paired-samples t-test: t(11) = 6.32, *p* = 5.68 × 10^−5^; Cohen’s dz. = 1.82). An exact Wilcoxon signed-rank analysis confirmed this result (W = 0, *p* = 0.000488; matched-pairs rank-biserial correlation = 1.00).

**Figure 2:**
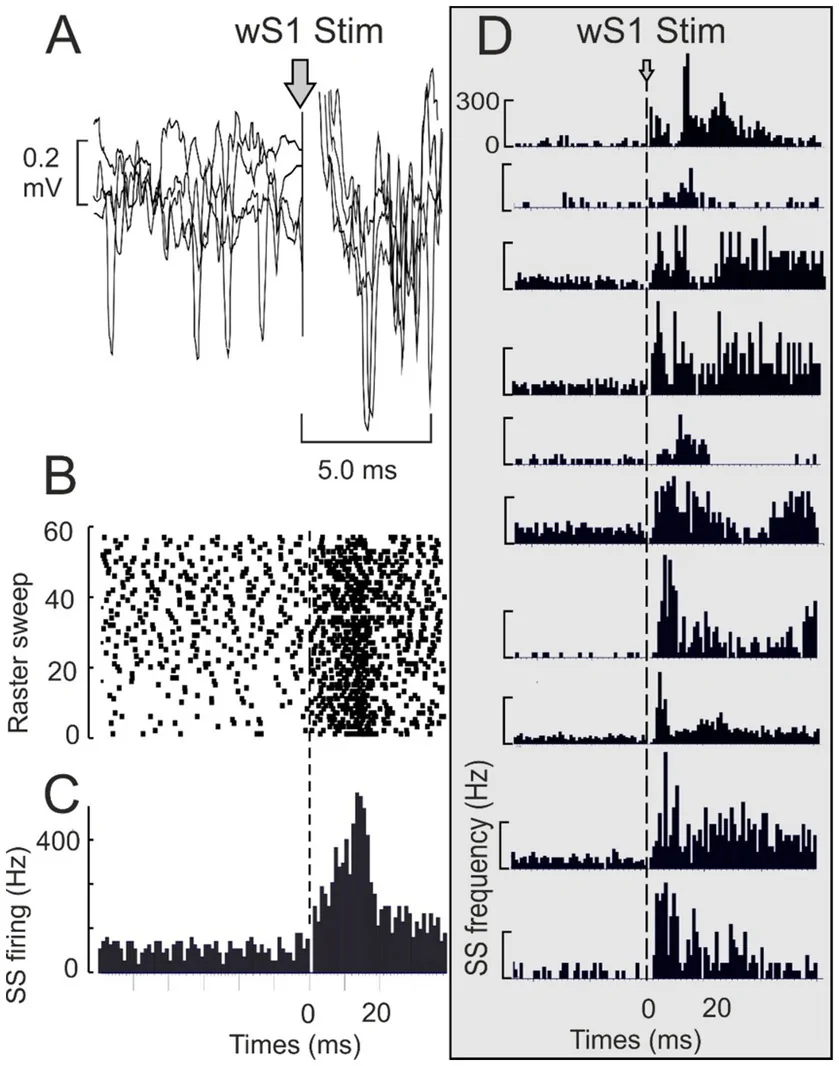
Recruitment of Purkinje cell simple spikes (SSs) by wS1 stimulation. **(A)** Four superimposed extracellular recordings from a representative Purkinje cell illustrating highly reproducible SS responses evoked by single-pulse wS1 stimulation (arrow). **(B)** Raster plot obtained during ~60 consecutive stimulation trials showing the trial-to-trial reliability of SS recruitment.**(C)** Peristimulus time histogram (PSTH) of SS firing for the same Purkinje cell shown in A and B, illustrating a marked increase in firing probability after stimulation. **(D)** PSTHs from 10 representative Purkinje cells illustrating the diversity of SS response profiles evoked by wS1 stimulation. The dashed vertical line indicates stimulus onset (0 ms). PSTH bin width of 1 ms.

**Figure 3:**
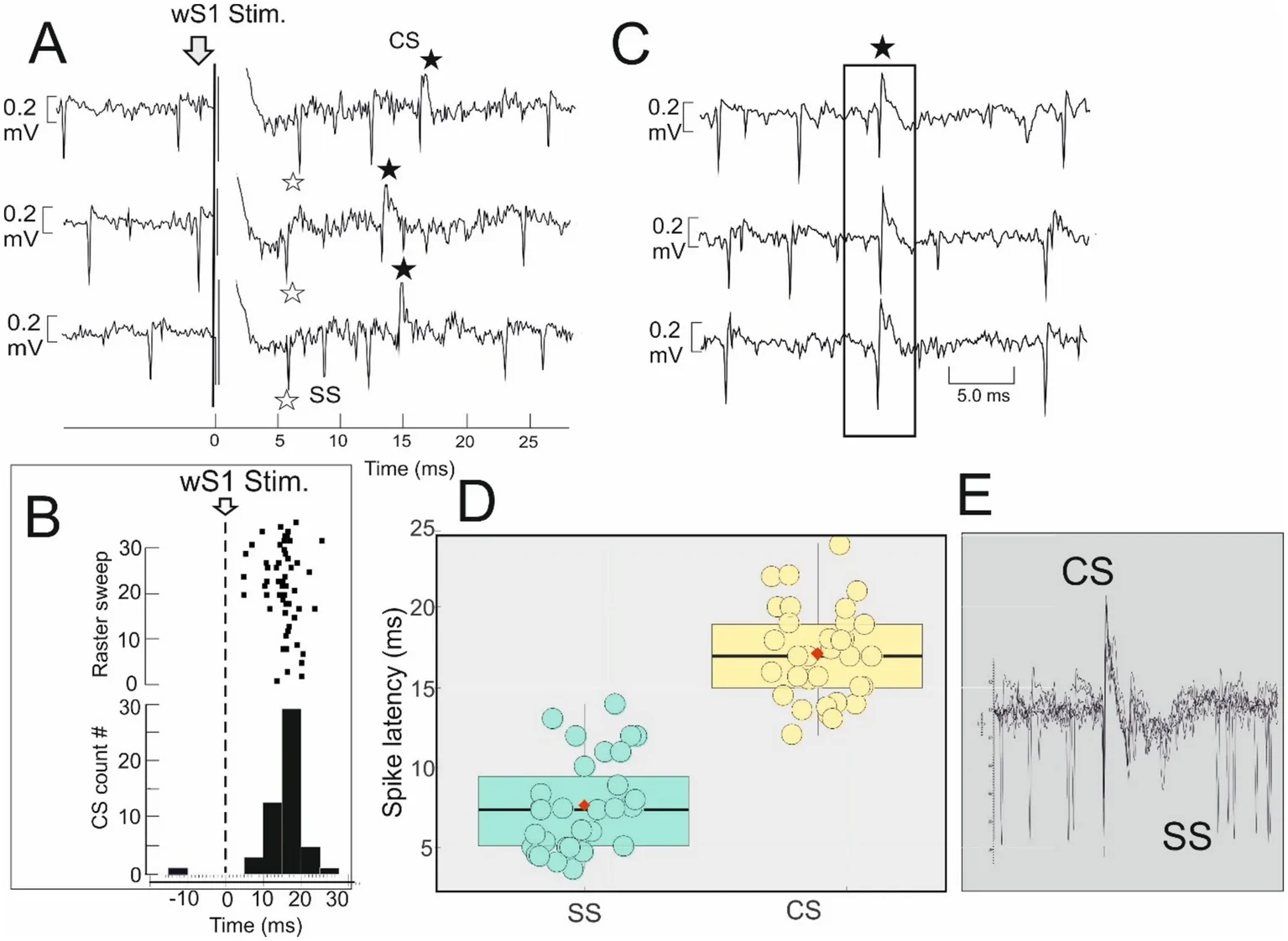
Temporal relationship between simple spikes (SSs) and complex spikes (CSs) evoked by wS1 stimulation. **(A)** Three representative trials showing SS (open stars) and CS (filled stars) responses recorded from the same Purkinje cell following wS1 stimulation (arrow). SSs were evoked at short latency (~6 ms), whereas CSs occurred at longer latency (~14–16 ms). **(B)** Raster plot and corresponding histogram illustrating the reproducible recruitment of CSs by wS1 stimulation (bin width of 5 ms). **(C)** Three spontaneously occurring CSs recorded in the absence of stimulation, demonstrating that evoked and spontaneous CSs displayed identical waveform characteristics. **(D)** Box plots comparing recruitment latencies of SSs (green) and CSs (yellow) across all recorded Purkinje cells. Each circle represents one neuron. **(E)** Superimposed traces aligned on CS occurrence illustrating the characteristic post-CS pause in SS firing, confirming Purkinje cell identification.

Figure 3A illustrates both the SS and CS firing during three wS1 stimulation trials where the SS were evoked at about 6 ms and the CS at around 15 ms (Figure 3B) in a representative PC. Three trials were presented in the absence of stimulation (Figure 3C) to show that the profiles of the spontaneous CSs were the same as those evoked by wS1 stimulation (Figure 3A). The mean onset latency of the earliest evoked SS-PC and CS-PC recorded on the 29 neurons in 18 mice were 7.4 ± 2.6 ms and 17.1 ± 2.8 ms, respectively, (Figure 3D). When the whisker pad was peripherally stimulated, the SS-PC and the CS-PC were recruited at the onset latency of 3.5 ± 0.5 ms and 11.3 ± 1.3 ms, respectively. PC response latencies were analyzed using a 2 × 2 repeated-measures design with spike type (SS and CS) and stimulation site wS1 and whisker-pad as within-animal factors. There was a significant main effect of spike type, with CS responses occurring later than SS responses (*F*(1,17) = 285.23, *p* = 4.65 × 10^−12^, partial η^2^ = 0.944). There was also a significant main effect of stimulation site, with longer latencies following wS1 than whisker-pad stimulation (*F*(1,17) = 118.62, *p* = 4.37 × 10^−9^, partial η^2^ = 0.875). The spike-type × stimulation-site interaction was not statistically significant (*F*(1,17) = 2.81, *p* = 0.112, partial η^2^ = 0.142). Holm-corrected paired comparisons showed that CS latency was longer than SS latency following both wS1 stimulation (mean difference: 9.6 ms, 95% CI: 7.5–11.7 ms; adjusted *p* = 7.93 × 10^−8^) and whisker-pad stimulation (mean difference: 7.9 ms, 95% CI: 7.2–8.5 ms; adjusted *p* = 6.12 × 10^−15^). Both SS and CS latencies were significantly longer following wS1 than whisker-pad stimulation indicating that the timing of the olivo-cerebellar module is different depending of the peripheral or cortical stimulation.

Although it was more difficult to recruit PC following wM1 stimulation than with wS1, (Figures 4A–E), mean PC SS latency was 7.7 ± 2.6 ms following wS1 stimulation and 9.9 ± 2.9 ms following wM1 stimulation. The mean paired difference was 2.2 ms (95% CI: 0.04–4.39 ms), indicating a modestly longer latency following wM1 stimulation (paired-samples t-test: t(15) = 2.17, *p* = 0.0462; Cohen’s dz. = 0.54). The latency difference was positive in 12 of the 16 mice. Sensitivity analyses yielded similar results (Wilcoxon signed-rank test: W = 30, *p* = 0.0494; exact paired-permutation test: *p* = 0.0487). At the animal level, mean PC CS latency was 17.1 ± 2.9 ms following wS1 stimulation and 23.1 ± 6.1 ms following wM1 stimulation. CS latency was longer following wM1 stimulation in 14 of the 16 mice. The median paired increase was 5.25 ms, and the difference was statistically significant (two-sided Wilcoxon signed-rank test with exact permutation: W = 6.5, *p* = 0.000458; matched-pairs rank-biserial correlation = 0.904). A paired-samples t-test confirmed this result (mean difference: 6.01 ms, 95% CI: 2.46–9.55 ms; t(15) = 3.61, *p* = 0.00258; Cohen’s dz. = 0.90).

**Figure 4:**
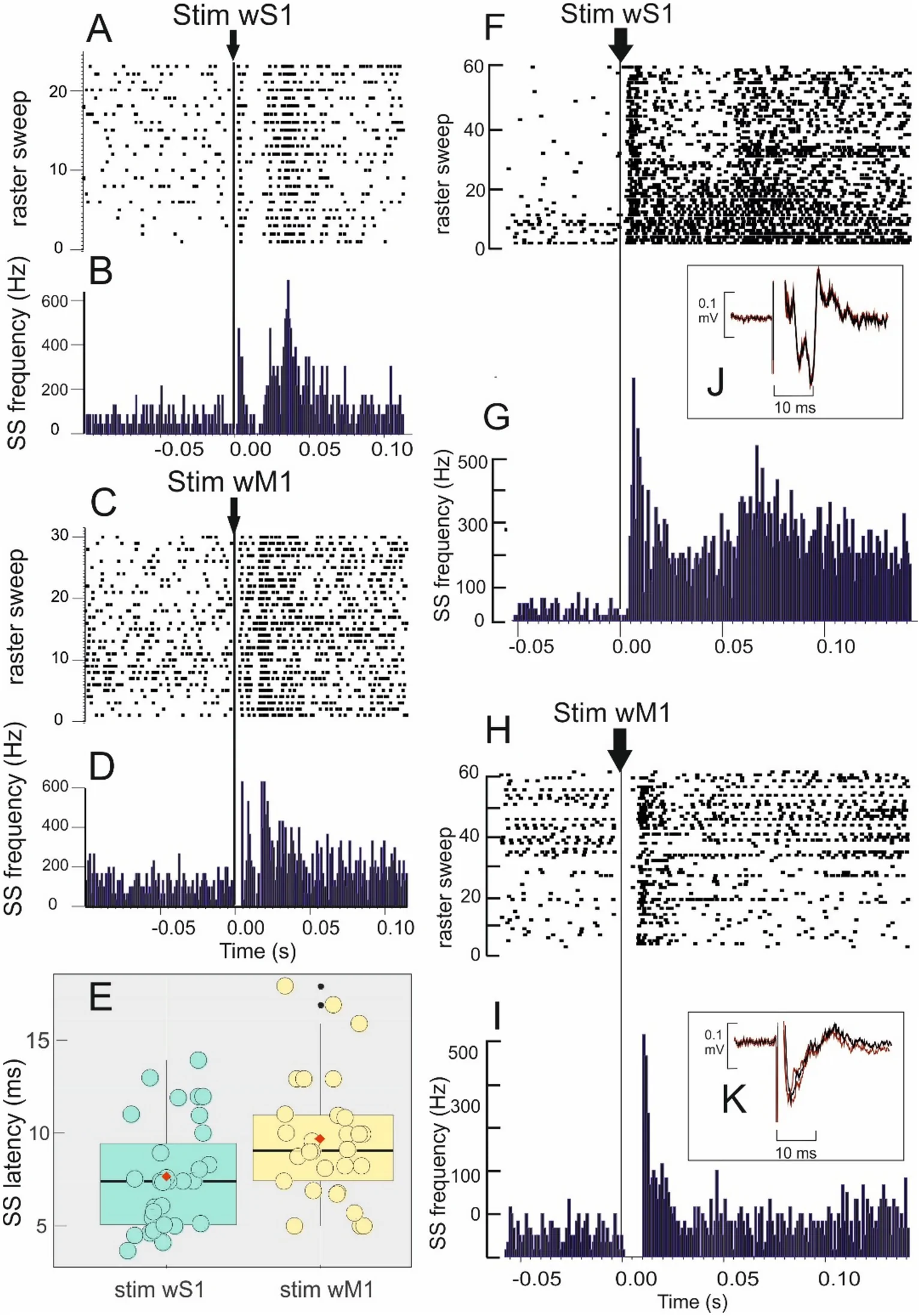
Comparison of Purkinje cell simple spike responses evoked by wS1 and wM1 stimulation. **(A,B)** Raster plot and PSTH (bin width of 0.15 ms) of a representative Purkinje cell during wS1 stimulation. **(C,D)** Raster plot and PSTH of the same Purkinje cell during wM1 stimulation. **(E)** Box plots comparing SS recruitment latencies evoked by wS1 (green) and wM1 (yellow) stimulation. **(F,G)** Raster plot and PSTH from a second representative Purkinje cell during wS1 stimulation, showing an early increase in SS firing followed by sustained activity. **(H,I)** Responses of the same Purkinje cell during wM1 stimulation, illustrating weaker and delayed recruitment. **(J,K)** Representative cerebellar LFPs simultaneously recorded in the Purkinje cell layer during wS1 **(J)** and wM1 **(K)** stimulation. wS1 stimulation evoked clear LFP responses, whereas wM1 stimulation produced little or no detectable LFP, indicating weaker synaptic activation of the cerebellar cortex.

The Figures 4A–D illustrates comparatively the firing behavior of a PC recruited by both wS1 and wM1. In this successful recruitment, the latency produced by wM1 was slightly different and the inhibition was less pronounced. However, the contrast between wS1 and wM1 was more often illustrated by the comparison made in Figures 4F–I. In this example, when wM1 was stimulated, the SS-PCs were briefly recruited at a latency of about 10 ms (Figures 4H,I), followed by a long lasting firing increase after 100 ms, versus a latency of about 5 ms followed by a second firing increase at about 50 ms when wS1 was stimulated (Figures 4F,G). In addition, a clear LFP presenting two negative components was evoked by wS1 stimulation (Figure 4J) while no LFP was induced by wM1 (Figure 4K).

### 3.5 Recruitment of DCN neurons

Figure 5 illustrates the firing behavior of a DCN neuron in response to wS1 stimulation. Four superimposed trials demonstrate a very short latency (2.8 ms) and low trial-to-trial jitter (0.45 ms) of the DCN response (Figure 5A top). These are consistent with the recruitment of a fast corticocerebellar pathway reaching the DCN with minimal synaptic delay. However, these temporal characteristics alone cannot establish monosynaptic connectivity, particularly because electrical stimulation may directly recruit corticofugal axons or fibers of passage. A second evoked response was obtained at the latency of 8.0 ms with a more important jitter of 1.75 ms, indicating the contribution of a polysynaptic circuit from wS1 to the DCN (Figure 5A bottom) or the contribution of the disinhibition exerted by the PC. These two types of evoked spikes may occur within the same trial or in separate ones, as illustrated here in the same DCN neuron for clarity. DCN firing frequency increased following wS1 stimulation in 17 of the 18 mice. At the animal level, mean firing frequency increased from 54.9 ± 41.9 Hz before stimulation to 89.1 ± 65.2 Hz (*n* = 28) after wS1 stimulation. This increase was statistically significant (two-sided exact Wilcoxon signed-rank test, W = 17, *p* = 0.00158; matched-pairs rank-biserial correlation = 0.80). The median paired increase was 24.9 Hz. The duration of the firing increase was about 350 ± 181 ms. For 12 out 28 DCN neurons, a relative decrease of DCN firing occurred at the mean latency of 19.6 ± 8.3 ms during a period of 25.9 ± 16 ms. At the animal level, mean DCN response latency was 5.6 ± 2.6 ms following wS1 stimulation and 4.7 ± 1.7 ms following whisker-pad stimulation. Although DCN responses were numerically later following wS1 stimulation, the difference was not statistically significant (two-sided exact Wilcoxon signed-rank test, W = 55, *p* = 0.113; matched-pairs rank-biserial correlation = 0.42). A paired-samples t-test yielded the same conclusion (mean difference: 0.8 ms, 95% CI: −0.2 to 1.9 ms; t(18) = 1.66, *p* = 0.114; Cohen’s dz. = 0.38).

**Figure 5:**
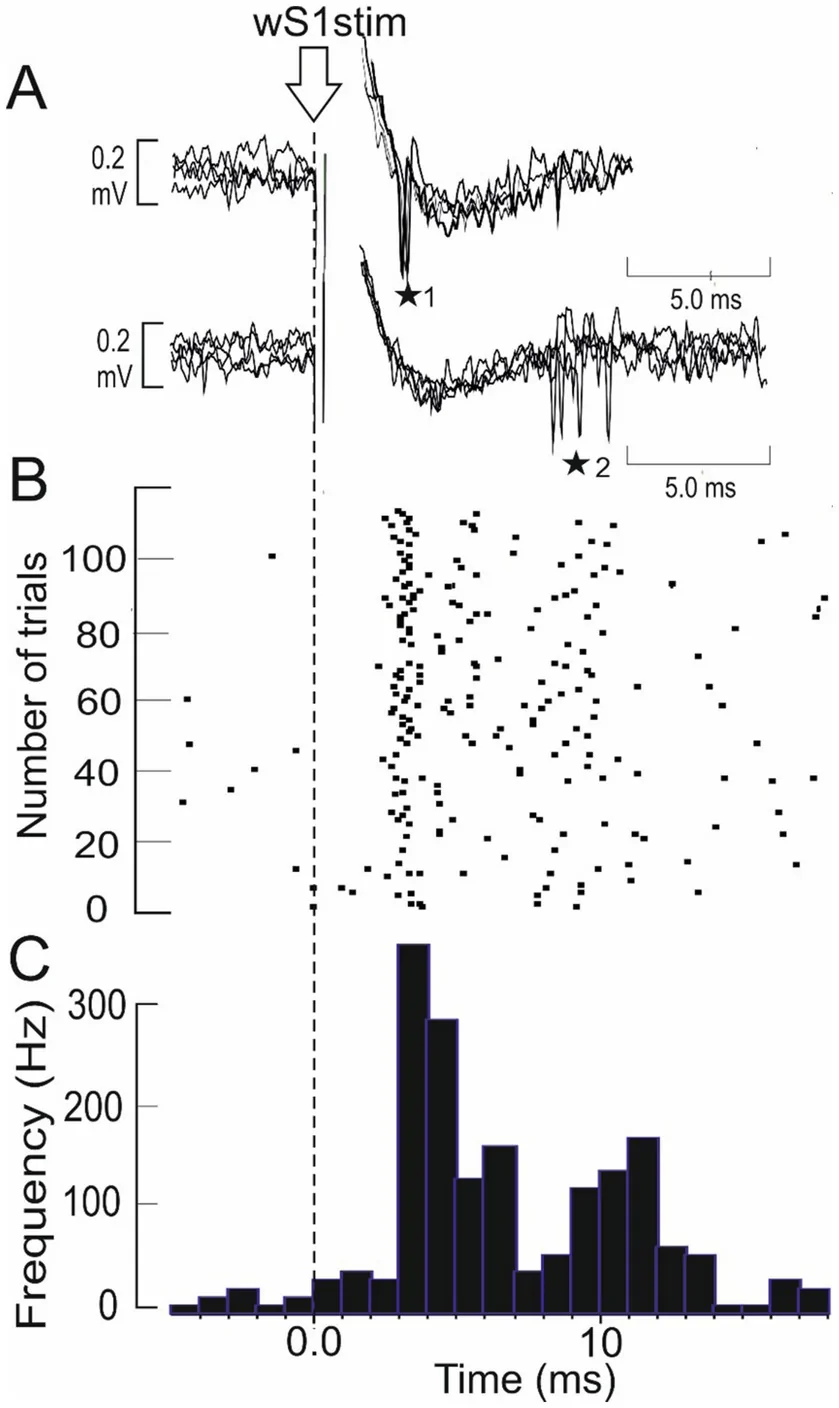
Recruitment of deep cerebellar nuclei (DCN) neurons by wS1 stimulation. **(A)** Superimposed traces from a representative DCN neuron showing two types of evoked responses following wS1 stimulation: an early, highly synchronized spike (top, star 1) and a later response with greater temporal variability (bottom, star 2). **(B)** Raster plot illustrating trial-to-trial variability of evoked DCN firing. **(C)** PSTH (bin width of 1.0 ms) of the same neuron showing stimulus-induced increases in firing probability. The bimodal profile reflects the presence of early synchronized responses and later responses exhibiting larger jitter. Dashed vertical line indicates stimulus onset.

As for the PC, wM1 stimulation (Figures 6B,D) was far less efficacious for evoking significant responses in the DCN than wS1 stimulation (Figures 6A,C). At the animal level, mean DCN response latency was 5.9 ± 2.6 ms following wS1 stimulation and 16.5 ± 3.9 ms following wM1 stimulation. DCN latency was longer following wM1 stimulation in each of the 17 mice included in the paired analysis. This difference was statistically significant (two-sided exact Wilcoxon signed-rank test, W = 0, *p* = 1.53 × 10^−5^; matched-pairs rank-biserial correlation = 1.00). The median paired difference was 12.00 ms. A paired-samples t-test confirmed this result (mean difference: 10.69 ms, 95% CI: 8.0–13.3 ms; t(16) = 8.61, *p* = 2.11 × 10^−7^; Cohen’s dz. = 2.09).

**Figure 6:**
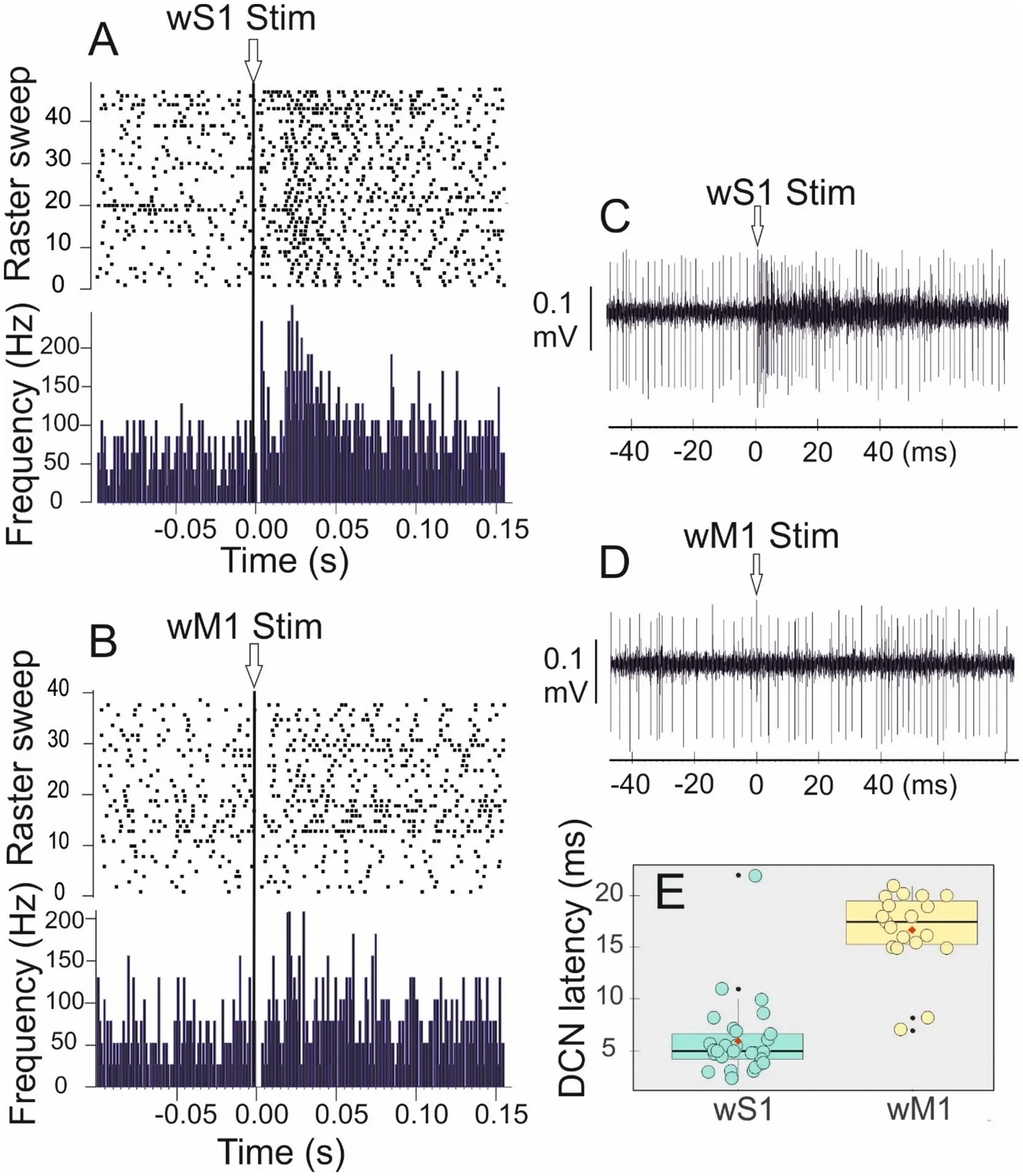
Comparison of deep cerebellar nuclei (DCN) responses evoked by wS1 and wM1 stimulation. **(A)** Raster plot and PSTH (bin width of 0.15 ms) from a representative DCN neuron during wS1 stimulation, showing an early synchronized response followed by a secondary increase in firing around 20 ms. **(B)** Raster plot and PSTH of the same neuron during wM1 stimulation. **(C)** Superimposed traces illustrating the short-latency response (~3 ms) evoked by wS1 stimulation. **(D)** Superimposed traces during wM1 stimulation showing the absence of the early response and the presence of only delayed activity. **(E)** Box plots comparing recruitment latencies of DCN neurons during wS1 and wM1 stimulation.

### 3.6 Recruitment of the Golgi cells

We recorded 40 well-identified Golgi cells in 20 mice presenting well characterized mean frequency of 10.4 ± 4.8 Hz and spike duration of 1.1 ± 0.1 ms. Among those cells, 25 were recruited by wS1 stimulation (Figure 7). This stimulation induced a strong evoked burst of Golgi cell spikes of about 9.5 to 15 ms in duration including 3–4 spikes (Figures 7B,C) at a mean latency of 6.0 ± 1.7 ms for the first spike of the short burst (Figure 7A). This burst reached a mean frequency of 300 Hz and was followed by long spiking silence of 33.4 ± 12.4 ms (Figure 7E). Following wS1 stimulation, the mean animal-level response latency was 5.7 ± 1.1 ms for Golgi cells and 7.7 ± 2.6 ms for SS PC. The mean paired difference was 2.0 ms (95% CI: 0.4–3.6 ms), indicating that SS PC responses occurred significantly later than Golgi-cell responses (paired-samples t-test: t(15) = 2.73, *p* = 0.0154; Cohen’s dz. = 0.68). A Wilcoxon signed-rank sensitivity analysis yielded the same conclusion (W = 20, *p* = 0.0130; matched-pairs rank-biserial correlation = 0.71).

**Figure 7:**
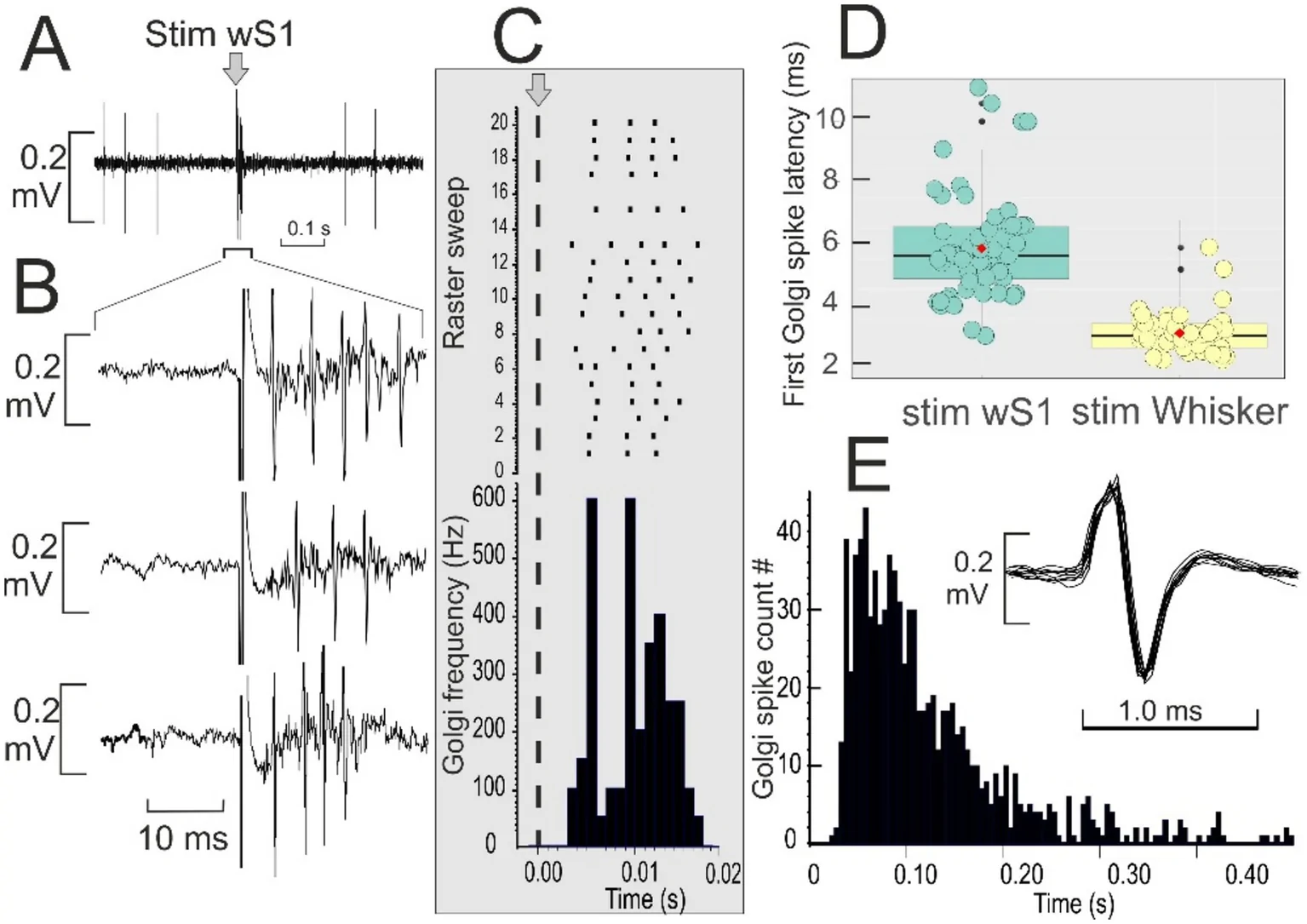
Golgi cell responses evoked by whisker pad and wS1 stimulation. **(A)** Representative extracellular recording showing that wS1 stimulation evoked a stereotyped burst discharge in a Golgi cell followed by a prolonged silent period. **(B)** Expanded traces illustrating the burst structure, typically composed of three to five spikes. **(C)** Raster plot and PSTH (bin width 0.5 ms) showing the reproducibility and timing of stimulus-evoked Golgi cell firing. **(D)** Box plots comparing the latency of the first evoked Golgi cell spike during wS1 stimulation and whisker pad stimulation. **(E)** Inter-spike interval distribution (bin width of 5 ms) the evoked Golgi cell burst together with superimposed spike waveforms used for neuronal identification.

Following whisker-pad stimulation, the mean animal-level response latency was 2.9 ± 0.4 ms for Golgi cells and 3.5 ± 0.6 ms for SSPC. The mean paired difference was 0.62 ms (95% CI: 0.22–1.02 ms), indicating that PC SS responses occurred significantly later than Golgi-cell responses (paired-samples t-test: t(16) = 3.29, *p* = 0.00465; Cohen’s dz. = 0.80). A Wilcoxon signed-rank sensitivity analysis yielded the same conclusion (W = 18.5, *p* = 0.00603; matched-pairs rank-biserial correlation = 0.76).

### 3.7 Recruitment of the IO neurons

The neurons recorded in the IO exhibited two main firing patterns: single-spike firing and bursting activity. The mean firing frequency of single-spike IO neurons was 20 ± 12.3 Hz (*n* = 16). In bursting IO neurons, the inter-spike interval (ISI) within bursts was 256.4 ± 205.3 ms, and the burst duration was 6.7 ± 2.6 ms (*n* = 10).

We compared, in randomly selected IO neurons and PCs, the intervals between spikelets of CSs in 10 PCs with the intervals between successive spikes within burst discharges of 10 IO neurons. The analysis is illustrated in Figure 8, in which four successive intervals were examined and their overall distributions compared using the Kolmogorov–Smirnov (KS) test. The first IO interval was 0.70 ± 0.15 ms, compared with 0.68 ± 0.15 ms for the first CS spikelet interval (*p* = 0.630, t-test; KS-d = 0.1818, KS-*p* = 0.6013). The second IO interval was 0.93 ± 0.40 ms, compared with 0.89 ± 0.29 ms for the second CS spikelet interval (*p* = 0.62, t-test; KS-d = 0.1916, KS-*p* = 0.4632). The third IO interval was 1.28 ± 0.69 ms, compared with 0.87 ± 0.28 ms for the third CS spikelet interval (*p* = 0.004, t-test; KS-d = 0.4965, KS-*p* = 0.0005). The fourth IO interval was 1.61 ± 0.86 ms, compared with 1.09 ± 0.58 ms for the fourth CS spikelet interval (*p* = 0.050, t-test; KS-d = 0.45, KS-*p* = 0.0377). No statistically significant differences were detected for the first two intervals between IO bursts and PC CSs. This support the existence of a physiological relationship between IO burst discharges and PC CSs.

**Figure 8:**
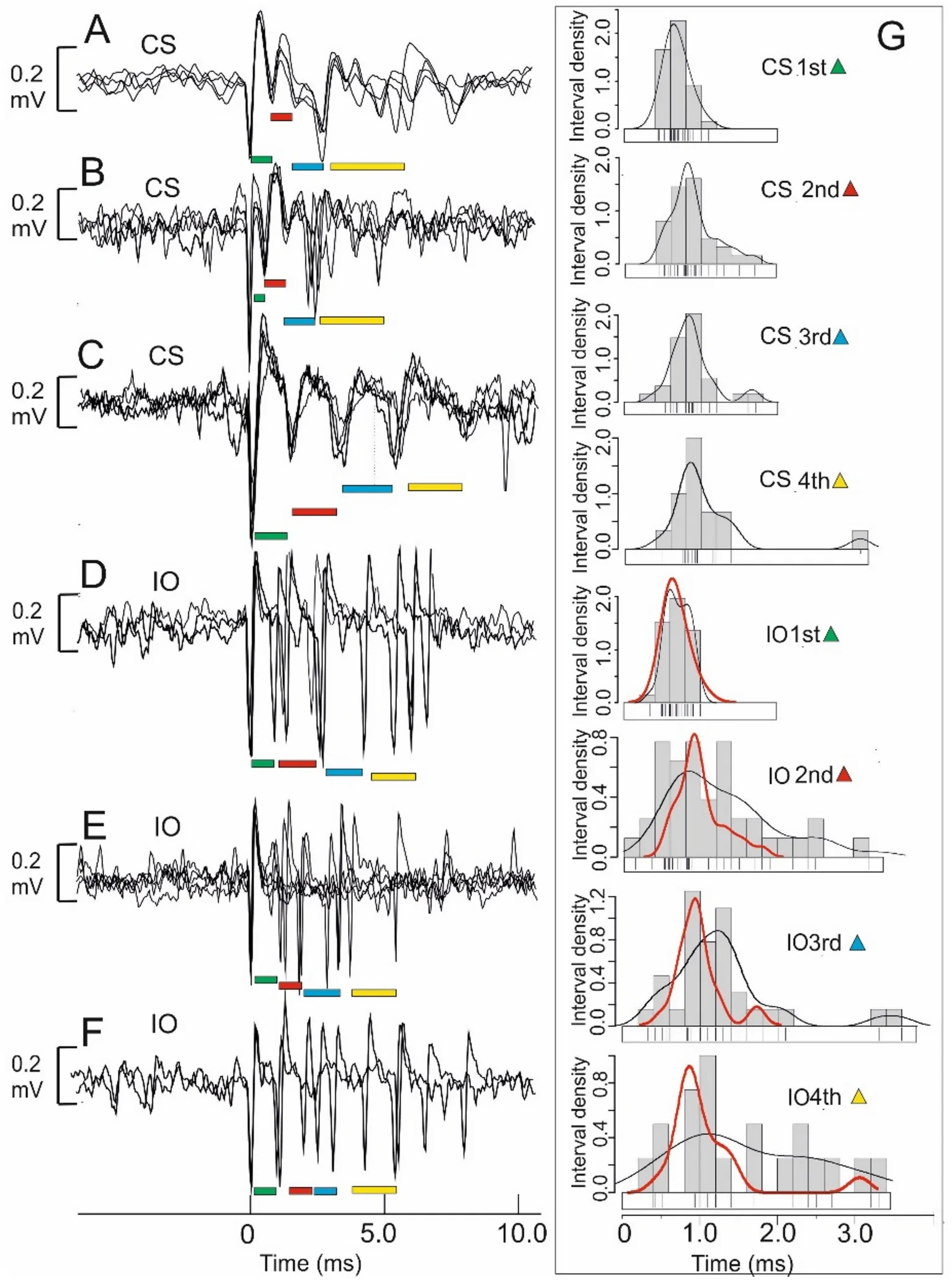
Comparison between inferior olive burst firing and Purkinje cell complex spike spikelets. **(A–C)** Representative CSs recorded from Purkinje cells illustrating the temporal organization of successive spikelets within a CS. **(D–F)** Representative burst discharges recorded from inferior olive neurons showing successive spikes within the burst. **(G)** Distribution of the first to fourth inter-spike intervals measured in Purkinje cell CSs (CS1st–CS4th) and inferior olive bursts (IO1st–IO4th), (bin width of 0.2 ms). Red curves represent the distributions obtained for Purkinje cell CSs superimposed on the corresponding IO interval distributions. Colored symbols identify corresponding interval orders. The first and second inter-spike intervals were not significantly different between Purkinje cells and inferior olive neurons, supporting a close physiological relationship between IO burst firing and Purkinje cell CSs.

The Figure 9 illustrated the recruitment of the IO neurons during the whisker pad (Figure 9A), the wS1 (Figure 9B) and the wM1 (Figure 9C) stimulation. IO response latency differed significantly among the three stimulation conditions (Friedman test: χ^2^(2) = 18.00, *p* = 1.23 × 10^−4^; Kendall’s W = 0.692; Figure 9D). At the animal level, mean latencies were 3.6 ± 0.5 ms following whisker-pad stimulation, 6.0 ± 1.5 ms following wS1 stimulation, and 7.8 ± 2.5 ms following wM1 stimulation. Post-hoc exact Wilcoxon signed-rank tests with Holm correction showed that IO latency was significantly longer following wS1 than whisker-pad stimulation (mean difference: 2.4 ms; W = 1, Holm-adjusted *p* = 0.000977; matched-pairs rank-biserial correlation = 0.978) and significantly longer following wM1 than whisker-pad stimulation (mean difference: 4.2 ms; W = 0, Holm-adjusted *p* = 0.000732; rank-biserial correlation = 1.00). Although IO latency was numerically longer following wM1 than wS1 stimulation, this difference did not reach statistical significance (mean difference: 1.7 ms; W = 23, Holm-adjusted *p* = 0.127).

**Figure 9:**
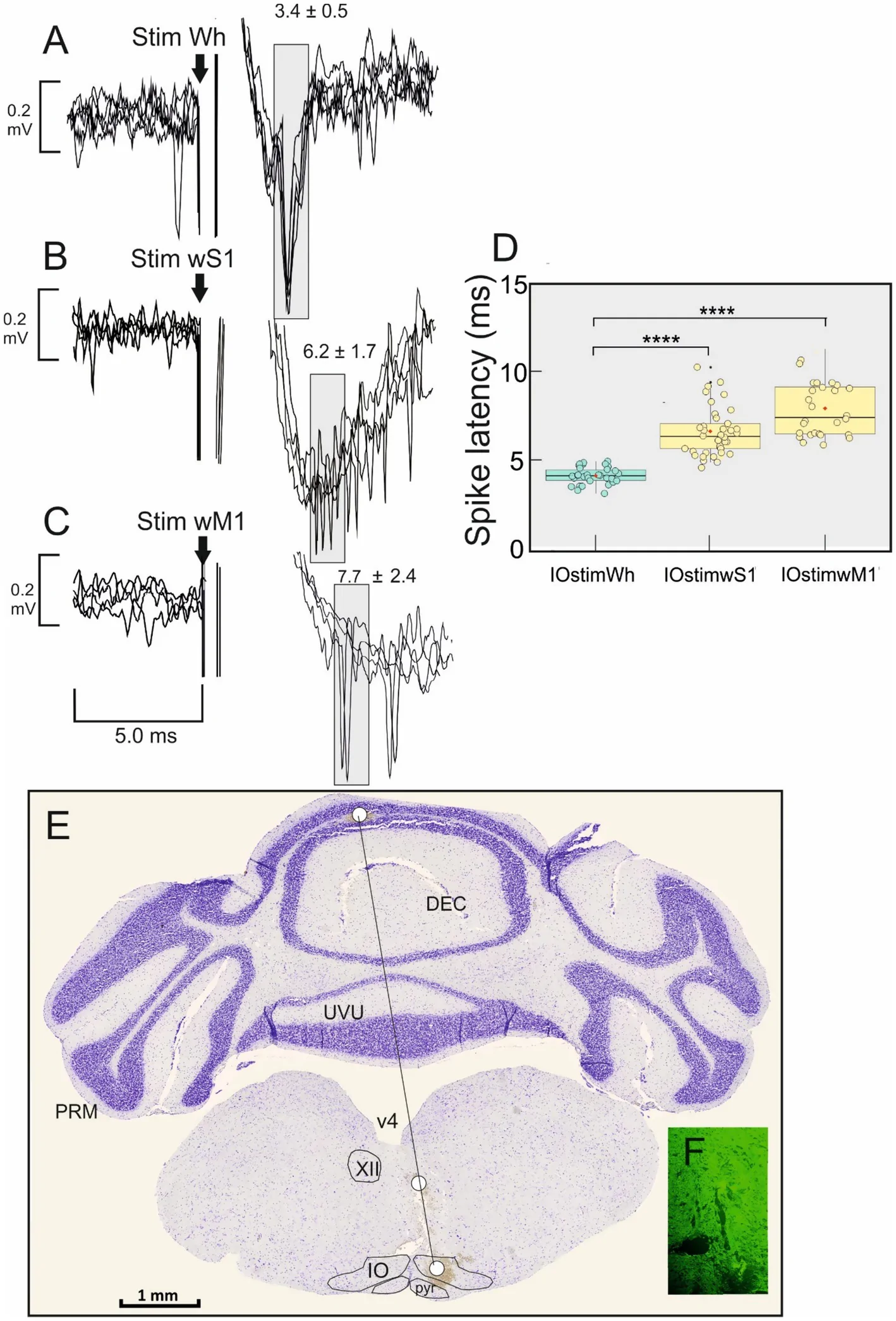
Recruitment of inferior olive (IO) neurons by peripheral and cortical stimulation. Spiking activity in the IO nucleus evoked by the stimulation of the whisker pad **(A)**, the wS1 **(B)** and wM1 **(C)**. The gray shaded rectangles highlight the time difference expressed by the mean ± SD for these 3 different types of stimulation, respectively. Dashed vertical lines indicate stimulus onset (0 ms). Whisker pad stimulation evoked highly synchronized short-latency responses, whereas wS1 and wM1 stimulation induced delayed responses with greater temporal variability (jitter). **(D)** Box plots comparing the onset latencies of IO neuronal responses evoked by whisker pad, wS1, and wM1 stimulation. Peripheral stimulation recruited IO neurons significantly earlier than either cortical stimulation. The four asterisks indicate the highly statistical significance. **(E)** Histological verification of the recording site in the inferior olive. Photograph of a brain slice passing through a recording tract showing the electrolytic lesion performed in the IO, 1 mm above and at the cortical entry point of the electrode trajectory. Representative micrograph showing the DiI-labeled electrode track and electrolytic lesion confirming the location of the recording electrode within the IO. Scale bar = 1 mm. DEC, declive; 4v, fourth ventricle; XII, hypoglossal nucleus IO, inferior olive; PRM paramedian lobule; UVU, uvula.

## 4 Discussion

### 4.1 Onset latency of whisker movement evoked by an electrical stimulus

The same electrical stimulation protocol was used to stimulate both the whisker pad skin and the wS1 and wM1 cortices. Although this type of stimulation is not physiologically natural, it offers the advantage of delivering an identical signal that induces axonal transmission in the form of action potentials, whose conduction velocity depends on axonal diameter and the number of synaptic relays encountered along the pathway. This approach allowed us to compare the latency of whisker movement onset and demonstrated that wM1 stimulation produced the shortest latency (~23 ms), followed by wS1 stimulation (~52 ms) and whisker pad stimulation (~55 ms). These latencies are consistent with those reported following optogenetic stimulation of the deep cerebellar nuclei (DCN; ~44 ms), Purkinje cells (PCs; ~40 ms), and the red nucleus (~35 ms; Bauer et al., 2025). Interestingly, the mean onset latency of whisker movements evoked by wM1 stimulation (~23 ms) closely matched the timing of the CSs elicited by wM1 stimulation. A similar temporal relationship was reported by Brown et al. (2024), who showed that, in the absence of electrical stimulation of wM1, CSs were time-locked to the onset of whisker movements. Although direct comparisons between non-specific electrical stimulation and highly selective optogenetic stimulation should be made with caution, the timing information provided by optogenetic studies may nevertheless help interpret the present findings and infer the final influence exerted by wS1 and wM1 on whisker movements.

Optogenetic activation of excitatory neurons in wS1 evoked a sustained retraction of the contralateral whiskers with a latency of 23.2 ms, whereas the same stimulation applied to wM1 induced rhythmic protraction with a latency of 36.5 ms (Sreenivasan et al., 2015; Sreenivasan et al., 2016). These latencies are consistent with those previously reported by Auffret et al. (2018) (33.8 ± 21.5 ms for wS1 and 61.5 ± 20.4 ms for wM1). However, these findings contrast with the present results, in which electrical stimulation of wM1 produced an earlier whisker movement (~23 ms) than stimulation of wS1 (~52 ms). One possible explanation is that electrical stimulation recruits inhibitory interneurons in wS1 more strongly than those in wM1, thereby delaying the motor output originating from wS1. However, this interpretation is difficult to reconcile with our observation that wS1 stimulation elicited earlier activation of cerebellar and inferior olivary targets than wM1 stimulation. Additional experiments directly comparing optogenetic and electrical stimulation of these cortical areas will therefore be required to resolve this apparent discrepancy.

### 4.2 Arrival of the peripheral signal in wS1, crus I-II and IO

It is interesting to report that despite the different pathways involved, the three structures (wS1, Crus I-II and IO) processed the input almost simultaneously, at about 3.0–3.5 ms after the stimulation of the whisker pad. The range of the stimulation-evoked LFP response latency in wS1 (ranging from 2.4 to 4.0 ms; Figure 1B) is consistent with the sensory response evoked by passive whisker deflection, which produces neuronal depolarization at about 5 ms in wS1 (Crochet and Petersen, 2006) and voltage-sensitive dye signal at a latency of 7.4 ms in awake head-restrained mice (Matyas et al., 2010). Similarly, the evoked LFP response latency (ranging from 2.2 to 3.9 ms) in Crus I matched the mean latency of PC firing (here, 3.47 ± 0.64 ms and 3.0 ± 0.5 ms in Márquez-Ruiz and Cheron (2012) following whisker pad stimulation.

Patch clamp recordings of PCs showed that SS firing changes often preceded whisking onset by 34.6 ± 7.9 ms, although with a broad range from −178 to 324 ms, indicating multiple timescales among Crus I (Chen et al., 2016). Likewise, PC firing preceded whisker protraction by ~15 ms during air-puff stimulation and by ~27 ms during spontaneous movement (Brown and Raman, 2018). These observations support the present results showing that the timing of the olivo-cerebellar loop depends on the nature of the input signal. When considering the temporal dynamics of the olivo-cerebellar circuit and its constituent neurons, it is noteworthy that both SS and CS responses of PC exhibited shorter latencies following peripheral stimulation (3.5 ms and 11.3 ms, respectively) than following stimulation of wS1 (7.4 ms and 17.1 ms, respectively) or wM1 (9.6 ms and 23.0 ms, respectively). A similar pattern was observed in IO neurons, whose response latencies were 3.4 ms following peripheral stimulation, 6.2 ms following wS1 stimulation, and 7.7 ms following wM1 stimulation. This temporal organization supports the notion that the contribution of the olivo-cerebellar circuit depends on the origin of the incoming signals, suggesting a specific functional role of this pathway in processing distinct sources of sensory and cortical information.

### 4.3 Similarity between IO neurons burst firing and PC CS spikelet

In whole-cell performed in anaesthetized mice by Khosrovani et al. (2007) four distinct firing patterns of IO neurons were described, with discharge frequencies ranging from 1 to 30 Hz, which is consistent with the mean firing frequency of approximately 20 Hz observed in the present study. These authors also reported different interspike intervals (ISIs) depending on the neuronal subtype, ranging from 139 to 641 ms, within which the present mean ISI of approximately 256 ms remains comparable.

The duration of the IO bursts observed here may be explained by the unique electrophysiological properties of IO neurons revealed by patch-clamp recordings and computational models (Mathy et al., 2009; De Gruijl et al., 2012) demonstrating the presence of subthreshold membrane potential oscillations upon which a sodium spike emerges, followed by an after-depolarization generating one to six spikelets. Such events could correspond to the spike sequences observed in our extracellular recordings. We observed a remarkably high degree of similarity between these intervals, supporting a physiological relationship between IO burst firing and PC CS spikelets. This finding confirms that the activity recorded here in the IO is likely to represent the source of the signals transmitted through climbing fibers and to contribute to the temporal organization of CSs. Although this analysis was performed using PCs and randomly selected IO neurons, the strong similarity in interspike timing between IO neuron bursts and CS spikelets further supports previous studies suggesting that the IO exerts a temporal imprint on the configuration of CS spikelets (Warnaar et al., 2015; Zang et al., 2018).

### 4.4 Early recruitment and firing behavior of Golgi cell

In the cerebellum, the earliest spiking activity (3.0 ± 0.7 ms) observed here in Golgi cell is consistent with the study of Vos et al. (1999) lead on anesthetized rats showing that the Golgi cell shortly responded to the whisker pad mechanical stimulation in a very similar way to that observed in the present case. An early spiking response arrived at the peak latency of 7 ms followed by a second one at 11 ms and a third one at 17 ms. The longer latency could be explained by species differences, the anesthetized sate and the mechanical stimulation.

Interestingly, the Golgi cells are one of the main inhibitory inputs exerted on the granule cells and are excited by feedforward action of the mossy fiber and granule cells feedback (Cesana et al., 2013). The mean synaptic delay between the GABAergic inhibitory postsynaptic current induced by electrical stimulation of Golgi cell axons presented a of the exponential decay time constant of about 40 ms (Dumontier et al., 2022). This could imply that a single pulse stimulation of wS1 can evoke at least 4 more synaptic peaks during the course of the first evoked synaptic event. In addition, the whisker pad electrical stimulation induced the same type of excitation pattern of the Golgi cell (short burst of 2–4 spikes at high frequency) as that induced by the electrical stimulation of wS1.

### 4.5 Firing behavior of the DCN neurons

It is important to note that DCN excitation (4.7 ms) occurs later than PC excitation (3.4 ms) when stimulation originates from the periphery, whereas following wS1 stimulation, DCN excitation (5.6 ms) precedes PC excitation (7.4 ms). This difference in timing may have important functional implications. Rebound disinhibition could account for DCN excitation during peripheral stimulation but is unlikely to explain DCN excitation following wS1 stimulation. However, it is possible that the direct action of the climbing fiber on the DCN, which has a latency of about 3.2 ms (Cheron and Cheron, 2018), can provide plasticity with a shorter delay than the IO but without the direct contribution of the online PC plasticity provided by CS-PC synapse. In addition, the connection delay between the DCN neurons and the red nucleus is about 1.1 ± 0.40 ms (Cheron et al., 2018). This could be integrated into the more general discussion about the existence of multiple sites of learning plasticity into the olivo-cerebellar circuitry (D’Angelo et al., 2009). The early response of the DCN to wS1 stimulation is consistent with the latency reported in the rat when the masseter nerve containing proprioceptive fibers was electrically stimulated, producing DCN responses ranging from 2 to 3 ms of latency (Tsutsumi et al., 2024). The present DCN firing behavior, characterized by two bursts, shortly followed by a longer one, is similar to the one recorded by Moscato et al. (2019) in response to air puff stimulation.

### 4.6 Functionnal implications of the different timing of the olivo-cerebellar loops and the forward dynamic model

The observation that wM1 stimulation induces a slower sequence of activation of the olivo-cerebellar circuit than that elicited by wS1 stimulation supports the possibility that efference-copy (EC) signals originating from wS1 and wM1 undergo distinct processing within the olivo-cerebellar circuitry. It may therefore be speculated that wS1 and wM1 provide two different delayed efference copies to the olivo-cerebellar system, which has been proposed to constitute the neural substrate of the forward dynamic model (Ramnani, 2006).

This interpretation further implies that the predicted sensory consequences generated by cerebellar output from the DCN are transmitted to wS1 and wM1 in a sequential manner. In addition, the comparison between the reafferent sensory feedback generated by sensorimotor output and the corollary discharge conveyed from the DCN to the IO could also occur in successive stages. In this framework, the IO, considered as a comparator, could generate distinct error signals at different time points. These signals would then be transmitted through the climbing fibers and processed through the interactive plasticity between SS and CS related input at the PC level.

The observation that the transmission delay between IO activation and the initiation of CSs varies according to the origin of the electrical stimulation (7.9 ms for peripheral stimulation, 10.9 ms for wS1 stimulation, and 15.3 ms for wM1 stimulation) further supports the existence of stimulus-specific processing within the dynamic network of the IO. This interpretation is consistent with the view that the IO does not merely transmit a uniform error signal, but may operate as a temporal comparator within the olivo-cerebellar system. IO neurons are electrically coupled through gap junctions and display intrinsic subthreshold oscillatory properties, providing a cellular substrate for temporally organized and synchronized climbing fiber activity (Mathy et al., 2009; De Gruijl et al., 2012). Such dynamic coupling may allow the IO network to transform incoming sensory, motor, and corollary discharge signals into temporally structured error signals distributed across cerebellar microzones. In this context, the different delays observed between IO activation and CS initiation after peripheral, wS1, and wM1 stimulation could reflect stimulus-specific processing by the oscillatory olivary network rather than simple differences in conduction time. This hypothesis is in line with the proposal that CS synchrony contributes to the temporal coordination and sequencing of motor commands (Welsh et al., 1995; De Gruijl et al., 2012; Lang et al., 2017; De Zeeuw and Romano, 2022). It is also in accordance with the idea that climbing fibers can convey temporally precise and graded instructive signals, rather than purely binary error messages, thereby allowing PC to adjust SS activity through the interaction between CS timing, SS dynamics, and synaptic plasticity. Thus, the IO may generate multiple, temporally shifted error signals that compare predicted sensory consequences with incoming reafferent information at successive stages of the olivo-cerebellar loop (Ju et al., 2019; Scheer and Prsa, 2025). Moreover, these different CS timings dependent on the origin of the input signal to the IO (Turecek et al., 2016) could be compatible with the recent model of Fernández Santoro et al. (2025), according to which the timing of CS plays a crucial role in the plasticity dynamics of the Upbound and Downbound PC behaviors. These short CS delays could also be compatible with the capacity of the PC-DCN-IO loop to integrate high frequency oscillations (160–200 Hz) of the cerebellum (Cheron et al., 2004; de Solages et al., 2008; De Zeeuw et al., 2008; Courtemanche et al., 2013). Interestingly, when the electrical pulse was applied in wS1 (Figure 6B), DCN spiking occurred at a similar time (~5.6 ms) to the onset of the Golgi Cell spiking (~5.7 ms) but earlier than the onset latency of the SS-PC firing (7.4 ms). Another important difference was the longer timing of the CS in response to the wS1 stimulation (range of 13.3 ms to 24 ms) in comparison with the CS timing when the whisker pad was stimulated (11 ms), which is in accordance with the results obtained by Márquez-Ruiz and Cheron (2012), who reported a range of 9.0 ms to 13.0 ms.

The present behavior of the PC and DCN neurons can be compared to the experiments of Brown and Raman (2018) in which these neurons were recruited by air puff stimulation. The onset of the DCN firing increase occurred at about 12 ms, while the onset of the SS PC firing increase occurred later, at about 15 ms, and was preceded by a short inhibition (Figure 6 of Brown and Raman, 2018). Such delayed PC activation relative to the DCN was reported here in the presence of the wS1 stimulation condition but not during the peripheral stimulation.

If the same sensory input activates the trigemino-cerebellar pathway and the cortico-ponto-cerebellar pathway, the PC could be successively activated with a delay of 14.5 ms for the SS and 18.2 ms for the CS. This CS latency corroborates the one reported by Van Der Giessen et al. (2008), with a latency of 29 ± 9 ms evoked by air puff stimulation in anesthetized whole cell recordings. If the wS1 initiates an independent command (not triggered by the sensory input) to the cerebellum, it is interesting to note that the latency of the CS evoked by wS1 stimulation occurs about 6.0 ms later that the ones evoked by the peripheral stimulation. In addition, a same delay of 6.0 ms occurs between the CS evoked by wS1 and the one evoked by wM1. Consequently, the same wS1 output signal providing an efferent copy of the future command cannot regulate actual whisker movements by passing to the olivocerebellar pathway because the wS1 induces a CS response latency of about 17 ms, which is too late for direct participation in the actual motor output. However, this is in accordance with the main role of the olivo-cerebellar loop acting as a comparator in the forward dynamic model. However, our results identify a temporal substrate potentially available for forward-model computation, rather than demonstrating the forward-model computation itself.

The different latencies and temporal recruitment patterns observed following wS1 and wM1 stimulation demonstrate that somatosensory and motor cortical inputs engage the cerebellar circuitry with distinct temporal dynamics. However, these differences cannot by themselves identify the functional nature of the transmitted signals. In particular, the longer latencies following wM1 stimulation may partly reflect the lower effectiveness of wM1 stimulation in recruiting cerebellar neurons, as well as differences in pathway excitability or synaptic organization. Therefore, our results do not directly demonstrate the transmission of an efference copy (EC) or the operation of a cerebellar forward model. Rather, we propose that the observed temporal organization is compatible with a framework in which sensory-related and motor-related cortical signals provide temporally distinct inputs to cerebellar circuits that could contribute to forward-model computation.

In accordance with a forward internal model, it is important to note that the cerebellar circuitry of Crus I-II is operatively active in relation to the processing of the peripheral input well before the arrival of the EC. Although the difference in DCN activation timing between whisker pad stimulation and wS1 stimulation was not statistically significant, activation occurred one millisecond earlier following whisker pad stimulation, leaving open the possibility for the DCN output initially activated by the peripheral input at a latency of 5.1 ms to send back cerebellar information about the periphery to the wS1 (Tanaka et al., 2020). This latter possibility is consistent with the forward internal models in motor control (Wolpert et al., 1995, 1998; Tanaka et al., 2020) and the consideration of the cerebellum as a predictor (Kakei et al., 2025).

The fact that the timing between wS1 and the DCN was about 6.0 ms suggests that wS1 can send its efference copy to the cerebellum and wait for the arrival of the dynamic forward output transmitted back from the DCN. The longer latency of wM1 in comparison with wS1 could be related to the relative weakness of wM1 stimulation in evoking cerebellar responses (Figure 10C) in this study. In addition, this results corroborates the fact that the magnitude of correlation between the pyramidal cells in wS1 and PC was higher than the correlation between wM1 and PC (Romano et al., 2025). The observed temporal difference between wS1 and wM1 could be in line with the existence of two parallel but behaviorally different cortical motor commands, one classically originating in the motor cortex (wM1) and the other in the somatosensory cortex (wS1; Sreenivasan et al., 2015). The wM1 projects via the reticular nuclei to the motoneurons (MN) of intrinsic muscles (protractor) situated in the ventrolateral (VL) facial nucleus, while the wS1 projects via the trigeminal nuclei to the MNs of extrinsic muscles (retractor) situated in the dorsolateral (DL) facial nucleus. Sreenivasan et al. (2015) demonstrated that optogenetic (ChR2) 50 Hz stimulation of wM1 evoked more rhythmic whisker movements than the same stimulation applied to wS1. A recent study (Maharjan et al., 2025) using rabies virus injection into the vibrissa muscle highlights the importance of motor commands issued from non-primary motor areas and proposes a new model where wS1 inhibits the MNs of protractor muscles, while in turn the wM1 inhibits the retractor muscles.

**Figure 10:**
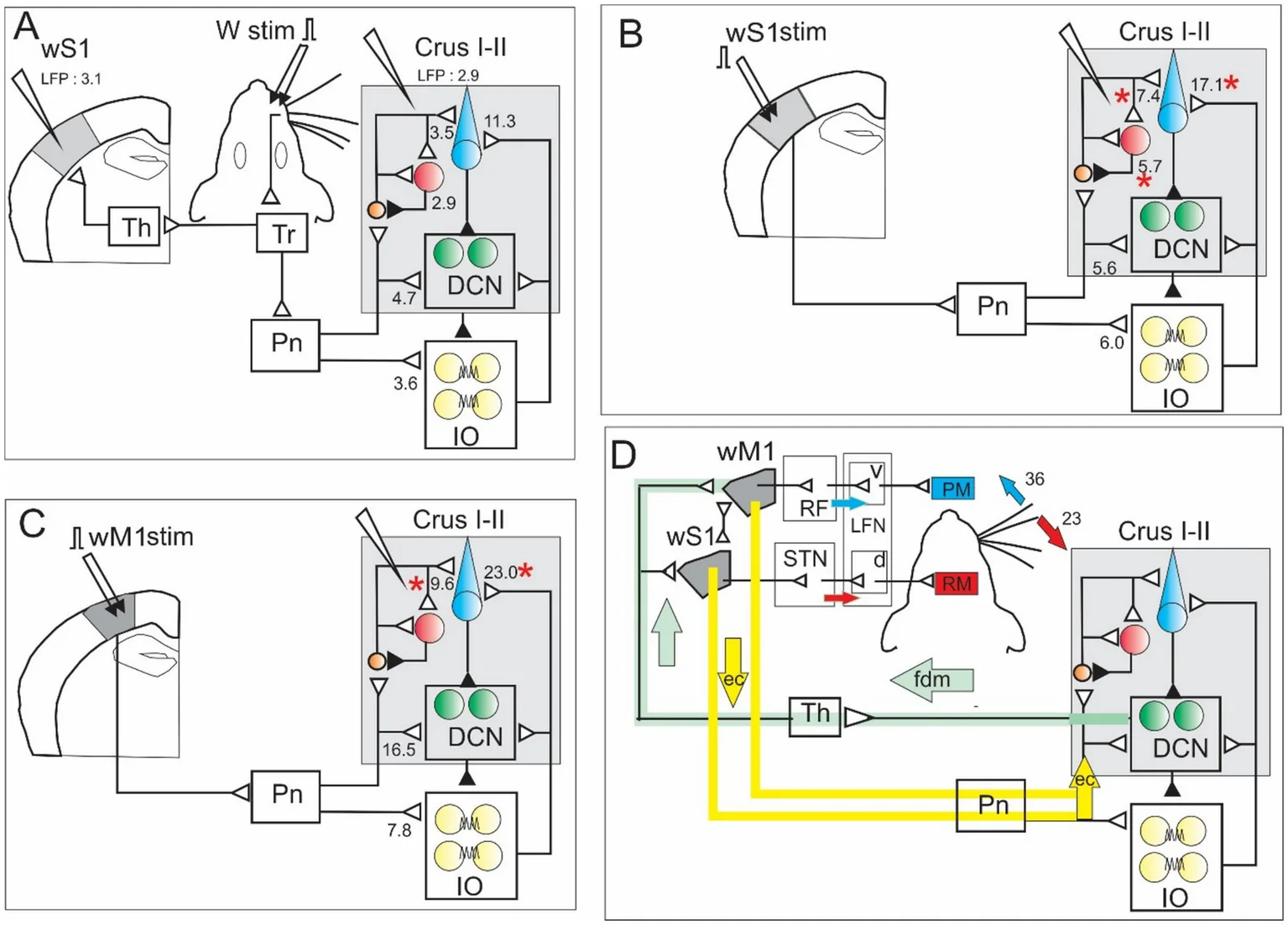
Proposed temporal organization of sensory reafference, efference copy, and cerebellar forward dynamic model during whisker movement control. **(A)** Schematic summary of the timing relationships obtained following whisker pad stimulation. Peripheral sensory inputs simultaneously activate wS1, Crus I–II, and inferior olive neurons at approximately 3 ms, followed by recruitment of deep cerebellar nuclei (DCN) neurons and Purkinje cell CSs. **(B)** Proposed sequence of events following wS1 stimulation. Cortical activation recruits the olivo-cerebellar loop with longer delays than peripheral stimulation, leading to sequential activation of Golgi cells, Purkinje cells, DCN neurons, and IO neurons. **(C)** Proposed sequence of events following wM1 stimulation. Motor cortical stimulation recruits cerebellar circuits at even longer latencies, consistent with additional processing associated with motor command elaboration. **(D)** Hypothetical model illustrating how sensory reafference and cortical efference copies may converge within the olivo-cerebellar network to generate forward dynamic models at multiple temporal scales. Solid arrows indicate experimentally demonstrated pathways, whereas dashed arrows represent hypothetical functional interactions. The Purkinje cells are represented in blue, the DCN neurons in green, the Golgi cells in red, the granule cells in orange and the inferior olive (IO) neurons in yellow. The spike latency onset evoked by the 3 different stimulations are, respectively, indicated closed to the corresponding symbolized synapses in ms. The theoretical and simplified pathways of the efferent copy directed to the cerebellum and the forward dynamic model output from the DCN are represented by yellow and light green arrows, respectively. The small red and blue arrows represent the commands and the related action of the retractor and the protractor muscles, respectively. The red stars indicate significant differences in timing activation between the wS1 and wM1 stimulation. DCN, deep cerebellar nuclei; ec, efferent copy; fdm, forward dynamic model; IO, inferior olive; LFN, lateral facial nucleus; Pn, Pontine nuclei; PM, protractor muscle RN, reticular nuclei; RM, retractor muscle STN, spinal trigeminal nuclei; Th, thalamus; Tr, trigeminal nucleus; v, ventral; d, dorsal.

## 5 Conclusion

From the possible scenarios arising from the timing relationships observed in the present study, together with those reported in the literature and within the framework of the widely accepted forward dynamic model (FDM), several possibilities emerge.

(1) If wS1 stimulation generates an efferent copy (EC) of a future motor command (yellow trace in Figure 10D), this signal would reach the DCN after approximately 6 ms. Assuming a 1 ms delay within the DCN and a further ~6 ms for the return projection to the cortex, the cerebello-cortical loop would require about 13 ms. This timing is compatible with the integration of a cerebellar FDM into both wS1- and wM1-related motor processing, given the whisker movement latencies observed following wS1 and wM1 stimulation (approximately 52 ms and 23 ms, respectively). If instead the EC originates from wM1, it would reach the cerebellum at approximately 16 ms, i.e., 10 ms later than the putative wS1-derived EC. The resulting wM1-cerebellar loop would still remain physiologically plausible, with the FDM signal returning at around 29 ms, leaving sufficient time for wM1 to generate the protractor command.
(2) If the FDM also requires the direct participation of PCs, an additional delay corresponding to PC activation by wS1 (7.4 ms) and PC-to-DCN transmission (approximately 1.5 ms) must be included. The total loop duration would therefore increase to about 22 ms. Such timing is too long to influence the initial wS1 output command but remains compatible with the transmission from wS1 to wM1 before the execution of the motor response.
(3) If the FDM further incorporates the olivo-cerebellar loop, the climbing fiber delay to PCs must be added. This would extend the loop duration to approximately 39.2 ms (22.1 + 17.1 ms), making it too slow to contribute directly to either the wS1- or wM1-related motor commands.
(4) Alternatively, if the IO acts as a comparator between the reafferent sensory consequences of movement and the corollary discharge encoding the FDM-predicted sensory outcome, the delayed arrival of the wM1-related signal through the olivo-cerebellar loop may permit comparison with the faster peripheral reafferent input at the level of the IO.

Taken together, these speculative considerations suggest the existence of multiple interacting loops linking the olivo-cerebellar network and the sensorimotor cortex. Within this framework, the rapid whisker-related peripheral input reaching the cerebellum (Figure 10A) could be integrated with the slower signals arising from wS1 (Figure 10B) and wM1 (Figure 10C). Such a temporal organization would provide several possible expressions of the FDM and could also accommodate the contribution of downstream brainstem pathways, including DCN projections to the red nucleus. Finally, the relatively slower transmission of wM1 signals within the cerebellar circuit may reflect the greater computational demands associated with the elaboration of the final motor command.

## 6 Limitations

The use of electrical stimulation of the whisker pad in place of more ecological stimulations, such as mechanical or air puff stimulation, may be considered a weakness. However, such type of single electrical pulse presented the advantage of being highly reproducible, inducing a well-synchronized afferent signals rapidly transmitted to the different central relays. In the same critical line, the electrical stimulations of the wS1 and wM1 were synaptically aspecific, producing the recruitment of excitatory and inhibitory neurons and related axonal tracts. However, the application of electrical pulses remained very effective for the direct stimulation of the axonal bundles, not only at the periphery but also in the cortex. If the final aim mainly concerns the initial timing of the firing recruitment and the basic temporal aspect of signal transmission between different regions, a controlled electrical pulse can be acceptable.

Because the present study focused on the timing of the olivo-cerebellar neurons, other important neurons in the circuitry between the sensorimotor cortex and the cerebellum and also in relation to the brainstem were not explored. These missing elements must temperated the present conclusion and will motivate new experiences.

## Data Availability

The raw data supporting the conclusions of this article will be made available by the authors, without undue reservation.
